# Federated learning with hyper-network—a case study on whole slide image analysis

**DOI:** 10.1038/s41598-023-28974-6

**Published:** 2023-01-31

**Authors:** Yanfei Lin, Haiyi Wang, Weichen Li, Jun Shen

**Affiliations:** China Telecom Research Institute, Guangzhou, 510000 China

**Keywords:** Cancer, Cancer screening, Biomedical engineering

## Abstract

Federated learning(FL) is a new kind of Artificial Intelligence(AI) aimed at data privacy preservation that builds on decentralizing the training data for the deep learning model. This new technique of data security and privacy sheds light on many critical domains with highly sensitive data, including medical image analysis. Developing a strong, scalable, and precise deep learning model has proven to count on a variety of high-quality data from different centers. However, data holders may not willing to share their data considering the restriction of privacy. In this paper, we approach this challenge with a federated learning paradigm. Specifically, we present a case study on the whole slide image classification problem. At each local client center, a multiple-instance learning classifier is developed to conduct whole slide image classification. We introduce a privacy-preserving federated learning framework based on hyper-network to update the global model. Hyper-network is deployed at the global center that produces the weights of the local network conditioned on its input. In this way, hyper-networks can simultaneously learn a family of the local client networks. Instead of communicating raw data with the local client, only model parameters injected with noise are transferred between the local client and the global model. By using a large scale of whole slide images with only slide-level labels, we mensurated our way on two different whole slide image classification problems. The results demonstrate that our proposed federated learning model based on hyper-network can effectively leverage multi-center data to develop a more accurate model which can be used to classify a whole slide image. Its improvements in terms of over the isolated local centers and the commonly used federated averaging baseline are significant. Code will be available.

## Introduction

In deep learning, the effectiveness and correctness of a model are highly dependent on the quality and quantity of training data available to a central server. The availability of Big-data and powerful computing units further the success of deep learning, which means it can benefit from large-scale training datasets with diversity from different sources and big models with great capacity^1^. This accelerated the application of deep learning technologies in domains such as finance^2^, healthcare^3–6^, transportation^7^, IoT^8^, e-commerce^9^, etc. User data is saved on a central server and used for subsequent training and testing processes, leading to a comprehensive ML model. In general, centralized deep learning-based approaches are linked with different factors, like computational power and the amount of time used, and most importantly, such centralized learning poses a threat to the security and confidentiality of user data.

Google introduced the notation of Federated Learning (FL) for the google keyboard in 2016 to collaboratively learn from different android phones^10,11^. Federated learning provides a way to protect user privacy by decentralizing data from central servers to end devices, allowing domains with sensitive and heterogeneous data to benefit from deep learning techniques^12^. This learning paradigm attracts research interests for two reasons: (1) not providing enough data to reside centrally on the server side (as opposed to traditional machine learning) due to restrictions on direct access to such data; and (2) using local data from edge devices (i.e. clients) to protect data privacy rather than sending sensitive data to servers where network asynchronous communication plays a role. In this sense, we “bring the code to the data instead of the data to the code”^13^. By preserving the privacy of local data, machine learning can leverage sufficient data from multiple domains to develop a robust model and increase its generality ability. In addition, since each center trains its model locally and only model parameters are communicated and shared through different centers, the burden on the centralized computational power can be alleviated remarkably.

Though FL is still in its infancy, some researchers from various institutions want to apply FL to ensure the privacy and security of user data. One of the areas that have benefited greatly from the federal framework is medical image analysis^14–16^. Because (1) medical data is **highly confidential** and is faced with legal and logistical obstacles (2) the transfer and storage of large-scale medical data **require a great hardware budget**. FL of medical data is a new research field in its initial stage, which has not won much trust in society. There are some early trails, though. Examples include large-scale multicentre genomic studies, attempts to establish globally scalable technologies, sharing of genomic data based on federal information infrastructure, the establishment of harmonized biobanking protocols, and material transfer agreements^17^. Chen *et al.* proposed Fedhealth to allow data aggregation using federated learning, followed by transfer learning to build comparatively individualized models^18^. Li *et al.* implemented and evaluated differential-privacy federated learning systems for brain tumor segmentation, and they concluded that there is a trade-off between improving model performance and reducing the cost of privacy protection^19^. The transfer and storage cost is peculiarly significant for large-scale computational pathology as 500 gigapixel whole slide images can be as large as an entire ImageNet^20^. By using FL, Andreux *et al.* aggregated computational pathology data to develop a Cox model for survival prediction^21^. Lu *et al.* introduced a differential-privacy technique for whole slide image classification using a weakly-supervised learning model without direct data sharing^20^.

We present a case study to demonstrate the feasibility and effectiveness of privacy-preserving federated learning using thousands of gigapixel whole slide images from multiple institutions. A new approach named hyper-MIL network of federated learning inherited from hyper-network^22,23^ is introduced to resolve privacy-preserving problems and model accuracy simultaneously.

## Method

### Preliminary

**Figure 1:**
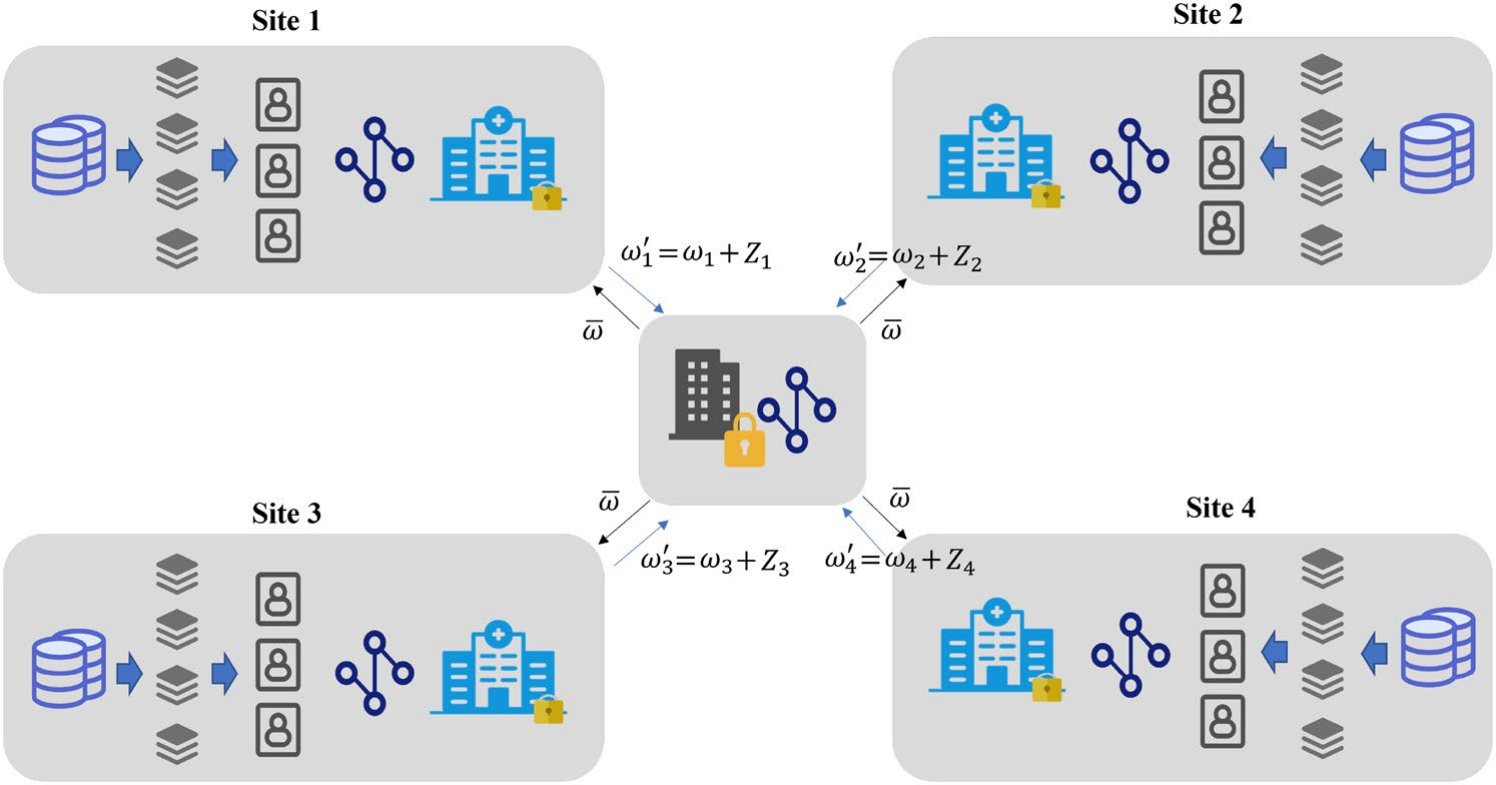
Federated learning with averaging algorithm. The centralized server sends averaged parameter $\bar{\omega }$ to each local client, and in return, each local client sends $\omega _{i}^{\prime }=\omega _i + Z_i$ back to the server, with $Z_i$ as the added noise.

Perhaps the most known and commonly used FL algorithm is FedAvg which was initially proposed by McMahan et al.^13^ and then extended for medical image analysis recently by Lu et al.^20^ and Adnan et al.^24^. It learns a global model by aggregating local models trained on independent identically distributed data, as shown in Fig. 1. Mathematically, FedAvg can be formulated as:

1 $$\begin{aligned} \min _{\omega \in R} f(\omega ) \quad \quad f(\omega )=\frac{1}{n} \sum _{i=1}^{n} f i(\omega ) . \end{aligned}$$

### Federated hyper-network Learning

Rather than training a central model by sharing the weights across different institutes, we train a hyper-network as an alternative to data sharing. Hyper-network^22,25^ is one kind of deep neural network that generates the weights for another bigger target network. The target network behaves the same as a usual supervised learning neural network, mapping the inputs to the desired labels. The hyper-network takes a set of inputs, named also embedding vector, with information about the structure of the weights, and generates weights for the layer.

**Figure 2:**
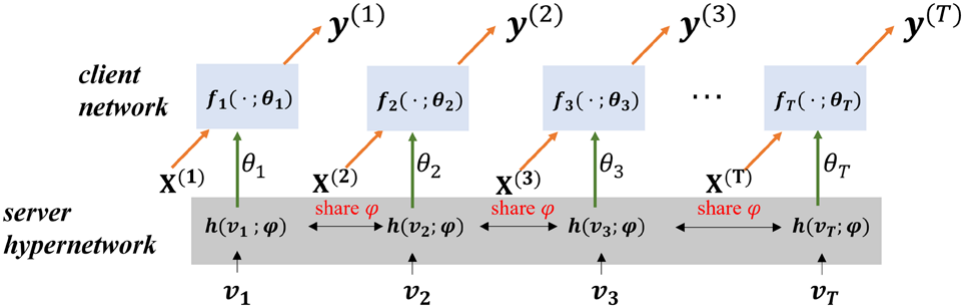
Hypernetwork at the server site share information across local sites implicitly through parameters $\varphi$, while generating each local parameter $\theta _i$ of the network with respect to embedding vector $v_i$.

As illustrated in Fig. 2, the hypernetwork $h(v_i;\varphi )$ deployed at the server site is parameterized by $\varphi$, taking the embedding vector $v_i, i\in \{1,..T \}$ as input to produce parameter $\theta _i$ for the $ith$ client network. The hypernetwork learns a family of neural networks $\{ \theta _i, i\in \{1,\ldots ,T\} \}$ simultaneously by sharing its parameter $\varphi$ across different clients. In this way, the parameter $\varphi$ of hypernetwork performs as a surrogate to exchange information. Embedding vector $v_i$ is a unique parameter of each local client that is jointly learned during end-to-end training, serving as a descriptor of a local client. In this regard, $v_i$ is client-specific and it provides client-dependent context and bias to hypernetwork to particular data distribution.

The objective of a federated hypernetwork is by minimizing all client networks with respect to their corresponding loss function:

2 $$\begin{aligned} {{\mathscr {L}}}_{\text{ hnet } } = \sum _{i=1}^{T} \left\| f_i\left( \textbf{x}^{(i)}; \theta _{i}\right) -y^{(i)}\right\| ^{2} \end{aligned}$$

where $\theta _{i} = h(v_i;\varphi )$, indicating that the data transferred from server to clients is independent of the model size of *h*. Thus, one can use an arbitrarily large hyper-network as long as it can enhance the overall performance.

The update of the hyper-network is achieved by calculating a chain rule of gradients, i.e.

3 $$\begin{aligned} \nabla _{\varphi } {\mathscr {L}}_{i}=\left( \nabla _{\varphi } \theta _{i}\right) ^{T} \nabla _{\theta _{i}} {\mathscr {L}}_{i} \end{aligned}$$

meaning that we should first calculate the gradient of ${\mathscr {L}}_{i}$ with respect to $\theta _i$, i.e., $\nabla _{\theta _{i}} {\mathscr {L}}_{i}$; then calculate the gradient of $\theta _i$ with respect to $\varphi$, i.e., $\nabla _{\varphi } \theta _{i}$. The parameter update of $v_i$ uses the same idea. As indicated by the formula, the local client optimizes the parameters $\theta _i$ using local training sample $\{ \textbf{x}^{(i)}, y^{(i)} \}$. The parameter $\theta _i$ is determined by the input unique embedding $v_i$ and shared parameters $\varphi$. In this way, the local client can maintain uniqueness using its training samples and enable parameter sharing. Thus, contradictory local update of the parameter is not a problem here.

**Figure 3:**
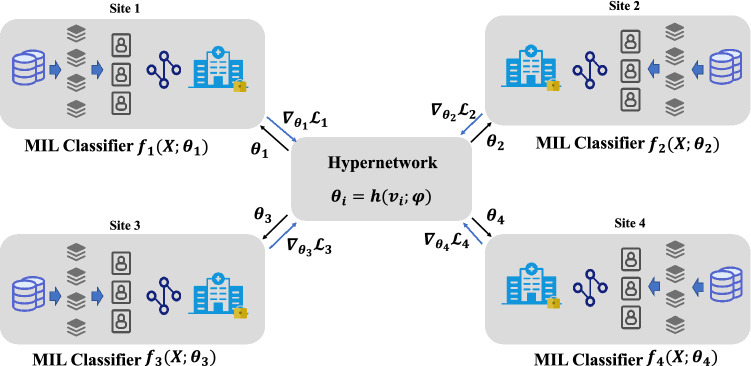
Federated learning with hypernetwork. Each local client has a MIL model, namely $f_1, f_2, f_3, f_4$, for whole slide image classification. On the server, a hypernetwork is used to generate the parameters of MIL classifiers. For parameter updates, the local client returns the gradient $\nabla _{\theta _{i}} {\mathscr {L}}_{i}$ over parameter $\theta _i$ to the server. Then, the gradient $\nabla _{\varphi } \theta _{i}$ over $\varphi$ is calculated to update $\varphi$. In this way, we can optimize the hypernetwork using all training data on local clients collaboratively.

With this approach, a central hyper-network model is trained to generate a set of models, one for each client. As shown in Fig. 3, this architecture provides effective parameter sharing across clients while maintaining the ability to generate unique and diverse individual models.

### Client classifier

Each local client learns a classifier to conduct whole slide image classification, which is formulated as a multiple-instance learning problem^26–28^. Since the whole slide image is a gigapixel (> $10,000\times 10,1000 pixels$), and only slide-level labels are used, we consider each whole slide image as a *bag* containing thousands of *instances* A bag is labeled as positive if any of its patches (instances) is disease-positive (e.g., with cancerous lesions). Otherwise, it is called a negative bag if all instances are negative.

Mathematically, let $B=\left\{ \left( \textbf{x}_{i}, y_{}\right) , i=1,2,\ldots ,K \right\}$ be a bag where $\textbf{x}_{i} \in \chi$ are instances with label $y_{i} \in \{0,1\}$. The label of *B* is determined by:

4 $$\begin{aligned} c(B)= {\left\{ \begin{array}{ll}0, &{} \text{ iff } \sum y_{i}=0 \\ 1, &{} \text{ otherwise } \end{array}\right. } \end{aligned}$$

The weakly supervised classifier first extracts patch-level features with a network *p*, then aggregates and examines these features with a classifier *g* to predict slide-level labels,

5 $$\begin{aligned} c(B)=g\left( p\left( \textbf{x}_{1}\right) , \ldots , p\left( \textbf{x}_{K}\right) \right) \end{aligned}$$

*p* is a pre-trained neural network, e.g., that maps patches into low-dimensional embedding vectors. For example, one commonly used setting is projecting a $256\times 256$ patch into a 1024 vector with ImageNet pre-trained ResNet50. Afterward, an attention-based network is introduced to score all embedding vectors according to their importance to task-specific labels^29^. The attention function is expressed as:

6 $$\begin{aligned} {\alpha _{k}}=\frac{\exp \left\{ \textbf{w}^{\top }\left( \tanh \left( \textbf{V} \textbf{x}_{k}^{\top }\right) \odot {\text {sigm}}\left( \textbf{U } \textbf{x}_{k}^{\top }\right) \right) \right\} }{\sum _{j=1}^{K} \exp \left\{ \textbf{w}^{\top }\left( \tanh \left( \textbf{V} \textbf{x}_{j}^{\top }\right) \odot {\text {sigm}}\left( \textbf{U x}_{j}^{\top }\right) \right) \right\} } \end{aligned}$$

where $\textbf{w}$, $\textbf{V }$, $\textbf{U }$ are learnable parameters; *K* is the total number of patches for a given bag; $\odot$ is an element-wise multiplication; ${\text {sigm}}(\cdot )$ is the sigmoid nonlinear activation function.

The final output of the client classifier is by weighting all patches:

7 $$\begin{aligned} y_{\text {pred}} = \rho \left( \sum _{k=1}^{K} \alpha _k x_{k}\right) \end{aligned}$$

where $\rho$ is a multilayer perceptron classifier.

Cross-entropy loss is minimized to train the client classifier:

8 $$\begin{aligned} {\mathscr {L}}_{\text {CE}} = \sum - y_{\text {gt}} \log y_{\text {pred}} \end{aligned}$$

We summarize the proposed federated hypernetwork for weakly supervised learning in Algorithm 1.
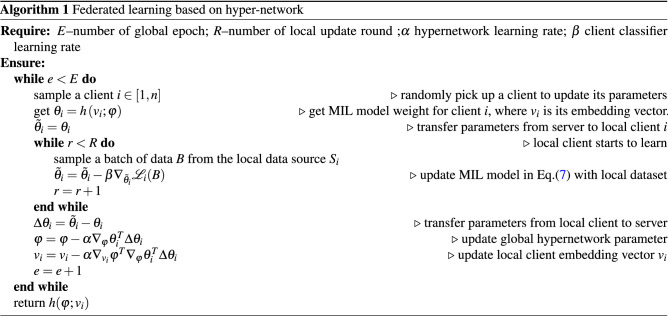


## Experiments

### Dataset

We employ two large-scale public data sets to verify our proposed approach.

*Prostate cancer dataset PANDA* Prostate cancer is the second most common malignant tumour in men all over the world. Prostate needle biopsy is the most reliable diagnostic method for patients suspected of prostate cancer. PANDA is the largest publicly available dataset of prostate biopsies to date with 10616 slides in total^30^. To simulate federated learning among multiple organizations, we arbitrarily divided the set of unique tissue source points into 5 non-overlapping, approximately equal-sized subsets, and grouped the data which are corresponding to each subset of tissue source sites, to serve as 5 distinct centres as shown in Table 1.

**Table 1:** Data split of PANDA.

	Site 1	Site 2	Site 3	Site 4	Site 5	All
Positive slides	1700	1686	1800	1760	1760	8706
Negative slides	380	367	400	370	393	1910
Total	2080	2053	2200	2130	2153	10616

*TCGA-NSCLC dataset* LUAD(Lung Adenocarcinoma ) and LUSC(Lung Squamous Cell Carcinoma), the two main sub-types of NSCLC(non-small cell lung cancer ), account for 65–70% of all lung cancers. To automatically classify these two main sub-types of NSCLC is an important step to help pathologists with more precise diagnoses. And We got 1043 hematoxylin and eosin (H &E) stained WSIs of lung cancer from TCGA. We divided the set of unique tissue source sites into 3 non-overlapping at random, approximately equal-sized groups as shown in Table 2.

**Table 2:** Data split of TCGA-NSCLC.

	Site 1	Site 2	Site 3	All
LUAD slides	167	178	186	531
LUSC slides	180	168	164	512
Total	347	346	350	1043

*Data preprocessing*The resolution of the whole slide image can exceed $10,000\times 10,000$ pixels. Therefore, instead of training with raw images, it is a standard practice to first crop the whole slide image into small patches with a size of $256\times 256$. About several thousand patches can be obtained for a single whole slide image. The dimension of image patches is further reduced by extracting 1024-dimension features with an ImageNet pre-trained RestNet50 model. The MIL classifier makes predictions with 1024-dimensional features.

### Evaluation metric

We can evaluate the federated learning performance by using diverse evaluation metrics from different views, i.e. the Area Under Curve of the ROC curve (receiver operating characteristic curve), classification error, balanced accuracy (bAcc), and F1 score

9 $$\begin{aligned} \begin{aligned} \text{ AUC }&= \int ^{b}_{a} y \cdot d x \\ \text{ Error }&= 1- \frac{{TP}+{TN}}{{TP}+{TN}+{FP}+{FN}} \\ \text{ recall }&=\frac{T P}{T P+F N} \\ \text{ precision }&=\frac{T P}{T P+F P} \\ \text{ specificity }&=1-\frac{F P}{F P+T N} \\ \text{ bACC }&=( \text{ recall } + \text{ specificity } ) / 2 \\ F_{1}&=2 \frac{ \text{ precision } \cdot \text{ recall } }{ \text{ precision } + \text{ recall } } \end{aligned} \end{aligned}$$

where *TP*, *TN*, *FP*, *FN* denote true positive, true negative, false positive, false negative.

### Implementation details

For each centre as described in Section "Dataset", we randomly split the data at local clients in the ratio of training:validation:test = 60:15:25 and then collect test data from all centres to constitute the global test set. Each centre trains a MIL model with its local training dataset. Also, we train a centralized model following the traditional way by collecting all training data at the server. For the federated learning paradigm, we use Federated Learning Average (FedAvg)^20,24^ as the baseline model to compare with. At the evaluation stage, all developed modes are evaluated with the global test set for a fair comparison.

Training setup. All models are implemented with PyTorch^31^. We use the Adam optimizer^32^ with $\beta _1=0.9$, $\beta _2=0.999$, $\varepsilon =1\times 10^{-8}$ and the base learning rate $=1\times 10^{-4}$. No weight decay is used in our models because rate constraints are inherently a regularization. We initialize the weights of models with a truncated normal distribution. All models are trained for 200 epochs for convergence. We ran all experiments 5 times and the final performance is averaged to get the mean value and the standard deviation.

## Results

### Results on PANDA

Table 3 shows the classification performance of all models including individual models, the centralized model, the federated learning model with naive averaging, and federated learning with the proposed hyper network design. In the table, each blank represents the mean and standard deviation. $\uparrow$ means the higher the better, and $\downarrow$ means the lower the better.

Not surprisingly, the centralized model achieves the best performance on AUC, Error, bACC, and F1 score because it is trained with a multi-center dataset and thus generalizes well on the multi-center test dataset. But the problem is obvious with this method: data security and privacy cannot be guaranteed in this setting.

In contrast, data can be protected if they are used only within the centre. Five local clients are trained with only a small amount of data and their performances are degraded significantly due to data limitations. Intuitively, one local model cannot “see” data from other centres and thus is not capable to learn diverse data distributions in practice. For this reason, local client models do not generalize well on the multi-centre global test set. For example, the AUC of local clients ranges from 0.912 to 0.929, greatly lags behind the centralized model of AUC=0.965.

**Table 3:** Results of classification on PANDA with or without Federated Learning.

	AUC $\uparrow$	Error $\downarrow$	$\textrm{bACC} \uparrow$	$\textrm{F} 1 \uparrow$
Site 1 only	$0.929\pm 0.004$	$0.167\pm 0.013$	$0.833\pm 0.027$	$0.815\pm 0.031$
Site 2 only	$0.928\pm 0.008$	$0.142\pm 0.038$	$0.842\pm 0.034$	$0.833\pm 0.021$
Site 3 only	$0.926\pm 0.014$	$0.148\pm 0.032$	$0.834\pm 0.013$	$0.841\pm 0.030$
Site 4 only	$0.915\pm 0.011$	$0.154\pm 0.023$	$0.825\pm 0.029$	$0.836\pm 0.019$
Site 5 only	$0.912\pm 0.009$	$0.165\pm 0.017$	$0.827\pm 0.037$	$0.830\pm 0.027$
Centralized	$0.965\pm 0.010$	$0.109\pm 0.022$	$0.911\pm 0.032$	$0.879\pm 0.009$
Federated Avg (baseline)	$0.945\pm 0.013$	$0.120\pm 0.025$	$0.878\pm 0.028$	$0.857\pm 0.025$
Federated HN (proposed)	$0.957\pm 0.009$	$0.117\pm 0.010$	$0.900\pm 0.021$	$0.866\pm 0.012$

Federated learning can address the dilemma of performance and data security. As the last two rows in Table 1 show, the performance is remarkably enhanced compared with local centres. For the baseline method of federated Avg, it improves the AUC from 0.929 to 0.945, Error from 0.142 to 0.120, bACC from 0.842 to 0.878, F1 from 0.841 to 0.857. But there is still room for further improvements as the critical design of federated Avg is naive average of all client model parameters. Our proposed method uses a hyper-network at the global server to generate weights for the client model and thus can potentially learn better model compared with naive averaging. As the last row in Table 1 shows, our proposed hyper-network model achieves 0.957 AUC, 0.117 Error, 0.900 bACC and 0.866 F1 score, surpassing the federated learning baseline method. This performance is approximating the upper bound of the centralized model which, however, considers no privacy preserving.

### Results on TCGA-NSCLC

Table 4 demonstrates the evaluation results on TCGA-NSCLC dataset. The AUC of local clients ranges from 0.886 to 0.895, and the baseline federated learning method can achieve 0.912. Compared to learning from a single-centre dataset, the model performance benefited greatly from training with multi-centre data using federated learning. Moreover, we found the proposed federated learning model of hyper-network achieves 0.920 AUC, 0.209 Error, 0.860 bACC, and 0.882 F1 score, generally competitive in performance with the centralized model where all training data is available.

**Table 4:** Results of classification on TCGA-NSCLC with or without federated Learning.

	AUC $\uparrow$	Error $\downarrow$	$\textrm{bACC} \uparrow$	$\textrm{F} 1 \uparrow$
Site 1 only	$0.881\pm 0.019$	$0.301\pm 0.034$	$0.827\pm 0.041$	$0.857\pm 0.030$
Site 2 only	$0.886\pm 0.032$	$0.332\pm 0.029$	$0.832\pm 0.033$	$0.841\pm 0.041$
Site 3 only	$0.895\pm 0.037$	$0.287 \pm 0.037$	$0.841\pm 0.031$	$0.855\pm 0.035$
Centralized	$0.932\pm 0.022$	$0.187\pm 0.034$	$0.875\pm 0.028$	$0.892\pm 0.037$
Federated Avg (baseline)	$0.912\pm 0.029$	$0.250\pm 0.024$	$0.850\pm 0.031$	$0.863\pm 0.042$
Federated HN (proposed)	$0.920\pm 0.030$	$0.209\pm 0.044$	$0.860\pm 0.039$	$0.882\pm 0.035$

### Visualization

Using the visualization tool from CLAM^28^, we can present the learned attention maps with hyper-network federated learning. In concrete, for an whole slide image with *n* image patches from client *i*, we can get the attention score $\alpha _k, k=\{1,2,...,n\}$ of each patch with the attention network in Eq. (6). The trained weight of attention network can be given by $\theta _i = h(v_i; \varphi )$.

As indicated in Eq. (7), the global feature is the weighted sum of all patches, and thus patches with higher attention scores can be pathologically more important. We thus normalize the attention score to (0, 1) and use it to draw a colormap. By overlaying the attention score and raw image, we can observe more interpretability results.

**Figure 4:**
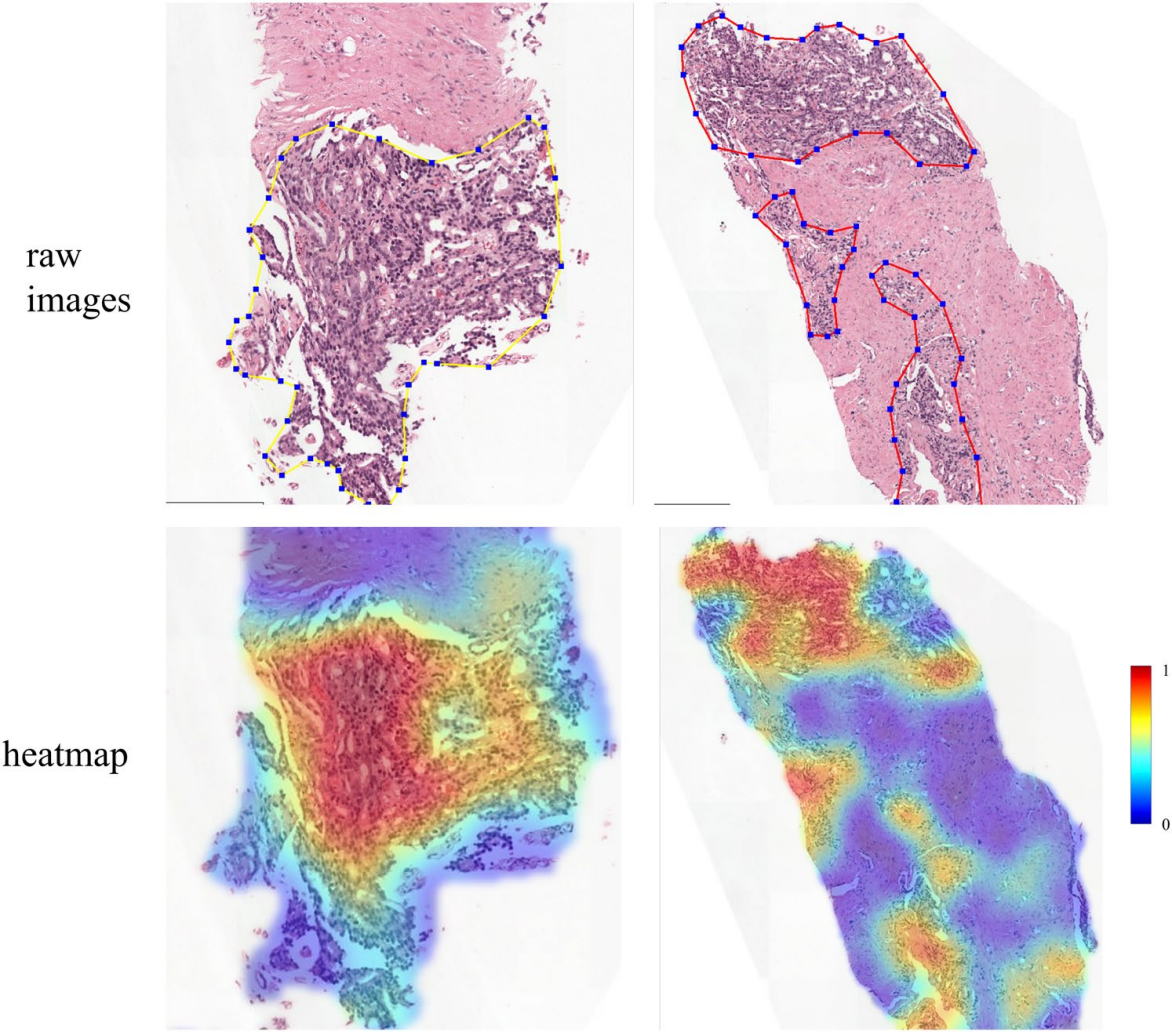
Heatmap visualization on PANDA dataset. The first line picture is the ground truth annotation from pathologists and the second line picture is the predicted heatmap from the MIL model. The lesions are highlighted in red color. The results indicate that our model is consistent with human pathologists for recognizing cancer lesions.

As shown in Fig. 4, we use two example images from the PANDA dataset. The most significant regions of a prostate whole slide image are cancerous lesions. The first row presents the raw images with pixel-level annotations, where the cancerous regions are delineated by pathologists. The second row is the views of heatmaps on tumor regions calculated by the attention network. The observation is that the regions are most strongly attended by the MIL model, and the localization of these regions consistently corresponds to the expert’s annotation. This can be interpreted by the findings that our proposed hyper network can effectively train each centre collaboratively to get clear and discriminative knowledge about cancerous lesions and normal tissue.

### Ablation study

In our proposed method, we use the attention-based MIL method in the framework of hyper-network federated learning. Alternatively, there are two commonly used baselines, i.e., max pooling and mean pooling. Max pooling aims to find the critical instance with the maximum score, and then use it as a global representation of the whole slide image with a much lower memory requirement. Mean pooling simplified the MIL problem by treating all the patches with the same label as the whole slide image. For inference, the model averages all the instances of the bag to obtain the global representation for classification.

As shown in Table 5, we conduct an ablation study using max pooling, mean pooling, and attention pooling in the framework of hypernetwork federated learning. Since max/mean pooling use no attention network, the communication cost between the server and clients is reduced compared with the proposed method. The results demonstrate that the classification performance of max/mean pooling degrades significantly. This is not surprising because max/mean pooling oversimplified the MIL problem that we only need to find one critical instance or we treat all instances equally. Both are not necessarily true. On the contrary, attention pooling automatically distinguishes the importance of the instance from the predicted labels.

**Table 5:** Ablation study using different MIL methods on PANDA dataset.

	AUC $\uparrow$	Error $\downarrow$	$\textrm{bACC} \uparrow$	$\textrm{F} 1 \uparrow$
Hypernetwork + Max Pooling	$0.919\pm 0.025$	$0.192\pm 0.045$	$0.810\pm 0.032$	$0.807\pm 0.051$
Hypernetwork + Mean Pooling	$0.905\pm 0.033$	$0.210\pm 0.031$	$0.778\pm 0.048$	$0.797\pm 0.040$
Hypernetwork + Attention Pooling (proposed)	$0.957\pm 0.009$	$0.117\pm 0.010$	$0.900\pm 0.021$	$0.866\pm 0.012$

### Discussion

Data protection and privacy preservation are bottlenecks of developing deep learning models for clinical usage. Considering that the data distribution at each local client can be heterogenous, it is more challenging to collaboratively train a model using all data. The proposed model is inspired by hypernetwork to produce network parameters for local clients while sharing training data collaboratively. It strikes a good balance between sharing data and maintaining the uniqueness of local clients. Thus, the performance is superior to the naive averaging strategy which treats all clients equally.

In the future, it would be an interesting direction to incorporate more cutting-edge techniques in the federated learning framework. For example, we can use self-supervised learning to extract features of the whole slide image instead of transferring the features from the ImageNet pre-trained encoder. One limitation of this work is that we have not yet scaled up to a number of local clients. More clinical data should be collected to evaluate the proposed federated hypernetwork. But it would require more effort and collaboration from multiple institutes, which is beyond the scope of this paper.

## Conclusion

Federated learning has been an emerging technique for learning from decentralized training data without explicitly sharing sensitive data to mitigate confidentiality and privacy issues. In this paper, we present a federated learning framework based on hyper-network by applying it to the whole slide image classification problem. Hyper-network deployed at the server centre learns to generate the MIL weights for each local client and in return, the training phase with local data provides model update information for the server model. We demonstrated that without transferring raw data from local client to server, our proposed hyper-network can collaboratively learn a good model that outperforms the commonly used federated averaging method. Visualization results on the whole slide image further provide evidence that our model learning is meaningful and pathologically import lesion in this federated learning framework.

## Data Availability

TCGA-NSCLC is publicly available at https://www.cancer.gov/about-nci/organization/ccg/research/structural-genomics/tcga. PANDA is available at https://www.kaggle.com/competitions/prostate-cancer-grade-assessment/data. Dataset generated during this study is available in this published article and also available from the corresponding author on reasonable request.

## Code availability

The code will be available upon acceptance.
